# Biochemical Effects of Long-Term Exercise on Oxidative Stress and Antioxidant Markers in Adolescent Female Athletes

**DOI:** 10.3390/children12070809

**Published:** 2025-06-20

**Authors:** Ömer Faruk Bilici, Dilara Erkan, Dan Iulian Alexe, Dragoș Ioan Tohănean, Canan Demir, Cristina Ioana Alexe, Virgil Ene Voiculescu, Muhammed Fatih Bilici, Héctor Fuentes-Barria, Ulas Can Yildirim

**Affiliations:** 1 Department of Sport Sciences, Institute of Health Sciences, Marmara University, Kartal, İstanbul 34865, Türkiye; obilici4@gmail.com; 2 Department of Physical Education and Sports, Institute of Health Sciences, Ankara University, Gölbaşı, Ankara 06830, Türkiye; derkan@ankara.edu.tr; 3 Department of Physical and Occupational Therapy, “Vasile Alecsandri” University of Bacău, 600115 Bacău, Romania; 4 Faculty of Physical Education and Mountain Sports, Transilvania University of Brașov, 500036 Brasov, Romania; 5 Department of Medical Services and Techniques, Van Health Services Vocational School, Van Yuzuncu Yil University, Tuşba, Van 65090, Türkiye; canandemir@yyu.edu.tr; 6 Department of Physical Education and Sports Performance, “Vasile Alecsandri” University of Bacău, 600115 Bacău, Romania; alexe.cristina@ub.ro; 7 Naval Tactics and Armament Department, Faculty of Marine Engineering, Mircea cel Batran Naval Academy, 900218 Constanta, Romania; virgil.ene@anmb.ro; 8 Department of Coaching Education, Faculty of Sport Sciences, Muş Alparslan University, Suvaran, Muş 49160, Türkiye; f.bilici@alparslan.edu.tr; 9 Vicerrectoría de Investigación e Innovación, Universidad Arturo Prat, Iquique 1110939, Chile; hefuentes_@unap.cl; 10 Facultad de Odontologia, Universidad Andres Bello, Concepcion 4030000, Chile; 11 Department of Coaching Education, Faculty of Sport Sciences, Sinop University, Osmaniye, Sinop 57100, Türkiye; ucyildirim@sinop.edu.tr

**Keywords:** oxidative stress, antioxidants, adolescent, female, athletes, exercise

## Abstract

Background: Adolescence is a critical period where exercise-induced oxidative stress is modulated by both training adaptations and hormonal changes, particularly the antioxidant effects of estrogen in females. However, data on how adolescent female athletes respond to long-term exercise remain limited. The aim of this study was to examine oxidative stress levels and some antioxidant defense parameters in adolescent female athletes who train regularly. Methods: The study included 20 adolescent female basketball players (16.65 ± 0.67 years; 165.50 ± 0.06 cm; 59.75 ± 5.50 kg) with at least three years of training experience and 20 non-athlete adolescent female participants (16.80 ± 0.69 years; 159.95 ± 0.04 cm; 60.15 ± 4.23 kg). Malondialdehyde (MDA), glutathione (GSH), and catalase (CAT) levels were analyzed by a spectrophotometric method using a UV/VIS spectrophotometer in blood samples taken from all participants, and the data were compared between the groups. Results: The results showed that MDA levels were significantly lower in the athlete group (*p* < 0.01; d = 4.78). In addition, CAT activity was significantly higher in athletes compared to non-athletes (*p* < 0.01; d = 7.81). However, no significant difference was observed in GSH levels between the groups (*p* > 0.05; d = 0.15). A strong negative correlation was found between MDA and CAT (r = −0.900). Conclusions: These findings suggest that prolonged exercise reduces oxidative stress and enhances catalase-mediated antioxidant defense in adolescent women. Increased CAT activity and decreased MDA levels support this effect, while stable GSH levels point to the role of compensatory mechanisms.

## 1. Introduction

Despite increasing global awareness of the importance of physical activity, recent global research indicate that 81% of adolescents and 27.5% of adults engage in a sedentary lifestyle, elevating the risk of chronic diseases, premature aging, and mortality by around 6–10% [1,2]. Sedentary behavior is associated with metabolic syndrome, cardiovascular disease, and neurological disorders, underscoring the critical necessity for preventive measures. Regular physical activity reduces these risks by enhancing cardiovascular, metabolic, and musculoskeletal health. The World Health Organization (WHO) advises a minimum of 60 min of moderate to vigorous aerobic exercise daily to enhance overall health. In addition, vigorous-intensity and muscle- and bone-strengthening activities are recommended at least three times per week to promote overall health [3].

Beyond general health promotion, exercise plays a fundamental role in regulating oxidative stress and enhancing antioxidant defenses—key processes for maintaining cellular balance [4]. Throughout various intracellular metabolic processes, molecules such as reactive oxygen species (ROS), reactive nitrogen species [5], and free radicals are generated. Oxidative stress occurs when the production of these reactive compounds and free radicals exceeds what antioxidant defense mechanisms can counteract [6]. It ultimately leads to cellular damage and can trigger the oxidation of vital biomolecules such as DNA, proteins, and lipids, causing many disease conditions [7,8]. In short, the oxidant–antioxidant balance, called the redox homeostasis of the organism, shifts in favor of oxidants. Redox homeostasis is not a balance in the thermodynamic sense; it is a stable state that manages vital biological processes such as mitochondrial energy production, signaling, and immune response [9]. Antioxidant molecules that constitute the antioxidant defense system include mechanisms that prevent free radical formation, cleanse free radicals, and repair damage caused by free radicals, serving redox homeostasis [8]. The antioxidant defense system is grouped under four headings as both enzymatic and non-enzymatic mechanisms, including catalase (CAT) and glutathione (GSH) [8,10]. These components from the first line of defense are essential for preserving redox equilibrium by counteracting the detrimental effects of reactive oxygen species (ROS), reactive nitrogen species [5], and free radicals [11].

While oxidative stress is typically linked to harmful consequences, moderate levels induced by exercise serve as beneficial stimuli for physiological adaptation. The effect of regular exercise on oxidative stress has similar characteristics to the hormesis theory. In toxicology, hormesis theory is the bell-shaped curve adaptation of biological systems to toxins and noxious stimuli. Hormesis is a bidirectional dose–response phenomenon that exhibits stimulatory or protective effects at low doses but becomes harmful at high doses. This situation is expressed by a non-linear response curve, usually described as a “J” or inverted “U” shape [12]. In summary, as the amount of the substance exposed increases, beneficial effects are initially observed, but after a certain threshold is exceeded, the effect decreases and becomes harmful. This feature is an important mechanism in understanding both the adaptive capacity of biological systems and the ways of coping with stress. According to the theory of hormesis, oxidative challenges provide cellular stimulation, preparing the organism for the next exercise session [13]. During exercise, increased oxygen consumption accumulates the production of reactive oxygen species and free radicals, developing oxidative stress. However, antioxidant defense mechanisms are stimulated to regulate the redox balance disrupted by increased reactive compounds. Accompanied by this stimulation, an increase in the effectiveness of defense systems is observed [13], while simultaneously improving the organism’s capacity to maintain redox equilibrium through the stimulation of antioxidant activity [14,15]. Biomarkers of oxidative stress, including malondialdehyde (MDA), are frequently assessed to determine oxidative stress levels in cells and tissues. MDA is generated when oxygen molecules and free radicals engage with polyunsaturated fatty acids in cellular membranes. First-line defense antioxidants, known for their inhibitory properties on the production of free radicals and reactive species [8], prevent oxidative stress and thus the generation of MDA. The increase in antioxidant reserves seen in adaptation to exercise-induced oxidative stress is critical for the suppression of MDA formation. Studies indicate that intense exercise immediately elevates oxidative stress indicators while temporarily diminishing antioxidant reserves [16,17]. Conversely, continuous exercise improves antioxidant capacity, diminishes oxidative stress, and augments physiological resilience [13]. A meta-analysis by de Sousa et al. [18] corroborates this viewpoint, indicating that regular exercise—irrespective of intensity, volume, or type—enhances antioxidant defenses and mitigates the immediate increase in pro-oxidant markers post-exercise. This long-term adaptation underscores the body’s ability to regulate metabolic stressors, ensuring a more stable redox balance and optimizing physiological functions in response to repeated oxidative challenges.

Emerging evidence also highlights the impact of exercise modality on oxidative balance in youth. For example, it has been reported that long-term aerobic exercises reduce oxidative stress by increasing antioxidant enzyme activity, while anaerobic-based exercises strengthen antioxidant defense systems in muscle tissue and blood [13]. In another study conducted with young and healthy male individuals, it was observed that gradual resistance exercises applied three days a week for 12 weeks increased antioxidant capacity and reduced oxidative stress index [19]. In addition, in a study conducted on elite athletes and examining different sports branches (cycling, taekwondo, rowing), different adaptations were detected in lipid peroxidation levels according to sports branch [20]. These results reveal that oxidative stress and antioxidant enzyme activity responses may vary depending on the type, intensity, and duration of exercise.

Sex-based physiological differences further complicate these responses. Sex-related differences in oxidative stress response are largely attributed to hormonal fluctuations. Estrogen exhibits well-documented antioxidative properties due to its phenolic hydroxyl group, which enables effective free radical neutralization, whereas the effects of testosterone on oxidative balance remain inconsistent [21]. Prior studies indicate that women exhibit greater resistance to exercise-induced oxidative stress than men, likely due to estrogen’s free radical scavenging capabilities [22,23]. Nonetheless, much of the current literature centers on adult male populations, leaving a knowledge gap concerning adolescent females. The effects of exercise on the antioxidant system may present variation not only in terms of sex differences, but also in the context of age and developmental stage. Adolescence is a critical period when the menstrual cycle, puberty-related hormonal shifts, and therefore the varying metabolic demands of training, introduce further complexity and differing biochemical responses to exercise from adults [24]. Additionally, it is not fully clear how the sport background influences oxidative stress parameters. Although Djordjevic et al. (2011) showed that increasing aerobic capacity is a determinant in reducing oxidative stress and increasing CAT activity [25], studies conducted on child and adolescent athletes such as Gougoura et al. (2007) and Santos-Silva et al. (2001) show that regular exercise can increase oxidative stress levels in this age group and that antioxidant defense mechanisms may not be fully developed [26,27]. These contradictory findings underscore the need for more targeted investigations, particularly in adolescent females, whose unique physiological characteristics warrant specific analysis. In this context, targeted investigation on the relationship between exercise-induced oxidative balance and antioxidant activity in adolescent female athletes is warranted.

Therefore, the objective of this study is to evaluate oxidative stress levels and the activity of key antioxidant markers (such as CAT and GSH) in adolescent female athletes with a history of long-term regular training. Accordingly, we assume that adolescent female athletes will exhibit lower oxidative stress and higher antioxidant levels compared to their sedentary peers and that there will be a strong relationship between oxidative stress markers and antioxidant defense parameters. Although the relationship between exercise and oxidative stress has been frequently examined in different populations in the literature, studies focusing on adolescent female athletes who have been regularly exercising for a long time are quite limited. Moreover, it has not been sufficiently elucidated how exercise-induced oxidative stress adaptations and antioxidant defense mechanisms are affected in this age group with a history of regular exercise for more than three years.

By considering both the physiological nuances of adolescence and the biological particularities of female athletes, this study fills an important gap in the literature on the effects of long-term exercise on oxidative stress and antioxidant defense and provides valuable information on sex-specific adaptations.

## 2. Materials and Methods

### 2.1. Participants

A cross-sectional study was conducted in accordance with the STROBE guidelines for observational research [28]. The study was conducted in accordance with the Declaration of Helsinki and approved by Ethics Committee of the of Mus Alparslan University Scientific Research, Türkiye (27 October 2021; approval no: 27305) and the ethical principles of the Declaration of Helsinki [29].

A total of 40 adolescent female participants, including 20 athletes and 20 non-athletes, aged 16 to 18 years, were recruited. All participants were students enrolled in secondary education and underwent anthropometric assessments and early-morning fasting blood sampling, followed by spectrophotometric analysis of oxidative stress biomarkers.

Inclusion criteria: female adolescents aged 16 to 18 years; free from chronic diseases; not using medications or ergogenic aids; and without musculoskeletal injuries in the past three months. Smoking and alcohol use were assessed through a self-report questionnaire administered prior to data collection, and only those who confirmed abstinence from both substances were included.

Exclusion criteria: diagnosis of any metabolic or systemic disorder; recent illness or infection; use of substances affecting oxidative metabolism; or incomplete data or failure to comply with pre-test instructions (e.g., fasting, rest, or activity restrictions).

Although the menstrual cycle phase was recorded to control for potential hormonal influences, this variable was not included in the statistical analyses, as previous studies indicate it does not significantly affect exercise-induced oxidative stress. Therefore, it was not considered a determining factor in the interpretation of results [30].

### 2.2. Training Protocols

The athlete group consisted of adolescent female basketball players with a minimum of three years of continuous training experience. These participants engaged in a structured training regimen supervised by certified coaches, comprising four sessions per week, each lasting approximately 90 min. The training included a balanced combination of technical drills, tactical instruction, aerobic conditioning, and strength-based exercises. Training intensity and workload were progressively adjusted according to individual performance and seasonal competition demands. In contrast, the control group included adolescents who did not participate in any form of structured or regular physical activity beyond standard school-based physical education classes. The distinction in physical activity levels between groups was corroborated through self-reported activity logs and validated by interviews with coaches and school staff.

### 2.3. Biochemical Sample Collection and Oxidative Stress Biomarker Analysis

Collection of blood samples: Blood samples of 3 mL were collected from the antecubital venous vein of all participants at a room temperature of 25 °C in a quiet room to ensure consistent environmental conditions, after resting for at least 30 min to minimize acute physiological fluctuations and ensure a true resting baseline, and transferred to biochemistry tubes. Participants were instructed to fast for at least 10 h prior to sampling to support circadian rhythm control and reduce dietary influences on oxidative biomarkers. Blood samples were centrifuged at 5000 rpm for 10 min. The obtained serum samples were stored at −80 °C until biochemical analyses were performed. To minimize the potential influence of circadian rhythms on oxidative stress biomarkers, blood samples were collected between 07:00 and 09:00 AM. Food consumption of all participants in the last 24 h was monitored and recorded.

### 2.4. Biochemical Analyses

All parameters were analyzed by spectrophotometric method with a UV/VIS spectrophotometer (Model T80+, manufactured by PG Instruments Limited, Leicestershire, UK). The device was routinely calibrated according to the manufacturer’s guidelines. All analyses were performed by the same trained technician to ensure consistency in handling and measurement. Each biochemical measurement was performed in triplicate to ensure reliability, and the average of the three readings was used for statistical analysis. The analyses were performed in the Central Biochemistry Laboratory of Van Yuzuncu Yil University, Faculty of Medicine. The tested parameters included malondialdehyde (MDA), glutathione (GSH), and catalase (CAT).

### 2.5. Malondialdehyde (MDA)

Malondialdehyde (MDA), a marker of lipid peroxidation, was measured using the thiobarbituric acid (TBA) method. In this method defined by [31], the formation of a pink complex by reacting with TBA at 95 °C and pH 3.4 conditions were taken as the basis. The absorbance value of the obtained complex at 532 nm wavelength was measured spectrophotometrically, and the MDA concentration was calculated. This was a manual method, and MDA concentrations were determined using a calibration curve based on known standards.

### 2.6. Glutathione (GSH)

The GSH level was assessed according to the method described by Beutler: A quantity of 800 µL of phosphate buffer was combined with 200 µL of serum. The initial absorbance (OD1) at 412 nm was recorded. A quantity of 100 µL of Ellman’s reagent was introduced into the same tube, followed by the measurement of the second absorbance (OD2) [32]. Standard curves and reagent blanks were used to ensure the accuracy of the results. Furthermore, highly technical procedural details were simplified while maintaining the core methodology.

### 2.7. Catalase (CAT)

Catalase (CAT) enzyme activity was measured spectrophotometrically at 240 nm according to the method of Aebi (1984) [33]. Phosphate buffer (pH 7.0) containing 30 mM hydrogen peroxide (H_2_O_2_) was used for the analysis. A quantity of 1.4 mL of 30 mM H_2_O_2_ and 0.1 mL of phosphate buffer were added to the control tube, while 1.4 mL of 30 mM H_2_O_2_ and 0.1 mL of serum sample were added to the sample tube. All reactions were conducted at a stable room temperature of 25 °C. Immediately after the reaction was started and every 30 s for a total of 2 min, the absorbance change was recorded spectrophotometrically at a wavelength of 240 nm. The timing of absorbance recordings was standardized to begin precisely after reagent mixing. Catalase activity was calculated based on the decomposition rate of hydrogen peroxide as ΔA/min (optical density change/min).

### 2.8. Bias

Despite efforts to ensure methodological rigor, this study is not exempt from potential sources of bias. First, the reliance on self-reported physical activity levels and lifestyle habits (e.g., smoking, alcohol consumption) may introduce reporting bias, although these data were cross verified through coach and teacher interviews. Second, while the inclusion of only female adolescents aimed to reduce variability, the absence of hormonal profiling or control for menstrual cycle phases—although justified based on existing evidence—may still represent a source of biological variation. Third, although sample size was determined a priori using power analysis, the relatively small number of participants may limit generalizability to broader adolescent populations. Additionally, since the study used a cross-sectional design, causality cannot be inferred from the observed associations between training status and oxidative stress biomarkers. Finally, despite matching groups on age and verifying training history, unmeasured confounders such as diet, sleep quality, or psychological stress may have influenced the outcomes.

### 2.9. Sample Size

The sample size was determined using G*Power software (version 3.1.9.4; developed by the Heinrich Heine University Düsseldorf, Germany), applying a two-tailed independent samples t-test. The calculation was based on a large, expected effect size (Cohen’s d = 0.80), as suggested by the previous literature, with a statistical power of 90% (1 − β = 0.90) and a significance level of α = 0.05 [34]. Results indicated that at least 17 participants per group were needed to detect statistically significant differences. To ensure adequate power and account for individual variability, the final sample included 20 participants in each group, which surpassed the minimum requirement established by the power analysis.

### 2.10. Statistical Analysis

The Shapiro–Wilk test was applied to test the assumption of normality of the data. The results indicated that all variables in both the athlete and non-athlete groups were provided the assumption of normality (*p* > 0.05), and the differences between the groups were determined using the Independent Samples *t*-Test. The statistical significance level was accepted as *p* < 0.05. Cohen’s d effect size was calculated to evaluate the magnitude of the difference between the groups and was reported with 95% confidence intervals. Effect size classification was made as follows: 0.20–0.49: Small effect; 0.50–0.79: Medium effect; ≥0.80: Large effect [34]. The Pearson Correlation Test was applied to evaluate the relationship between oxidative stress marker (MDA) and CAT/GSH-mediated antioxidant defense. The effect size of the correlation was classified as 0.10–0.29: Small; 0.30–0.49: Medium; ≥0.50: Large [35]. All statistical analyses were performed using IBM SPSS Statistics 25.0 (IBM Corp., Armonk, NY, USA) software, and results are reported in the format of mean ± standard deviation (Mean ± SD).

## 3. Results

The final sample consisted of 40 adolescent females, evenly divided into an athlete group (n = 20) and a non-athlete group (n = 20). The mean age was similar between groups (athletes: 16.65 ± 0.67 years; non-athletes: 16.80 ± 0.69 years), with no statistically significant difference (*p* = 0.49) and a medium effect size (d = 0.45).

Anthropometric analysis revealed significant baseline heterogeneity between groups in terms of height and body mass index (BMI). Athletes were significantly taller (165.50 ± 6 cm) than non-athletes (159.95 ± 4 cm), with a large effect size (d > 0.80) and a highly significant difference (*p* < 0.001). Likewise, BMI was significantly lower in the athlete group (21.81 ± 1.50 kg/m^2^) compared to non-athletes (23.51 ± 1.60 kg/m^2^), also with a large effect size and *p* < 0.001. These differences indicate a non-trivial anthropometric divergence at baseline (Table 1). No significant differences were found in body weight between groups (*p* = 0.79), despite a slightly lower mean weight in the athlete group.

**Figure 1:**
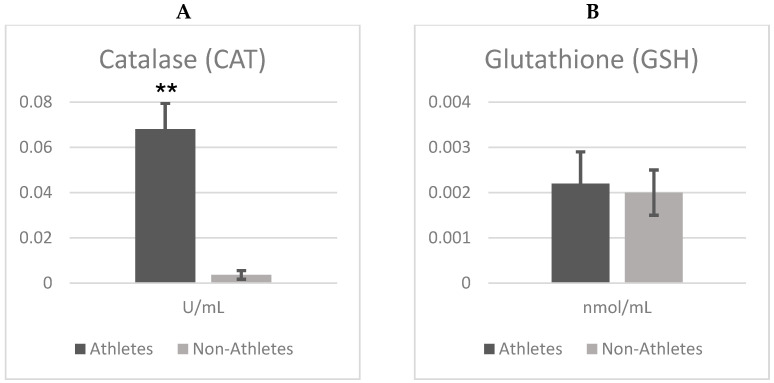
(**A**,**B**). Comparison analysis of CAT and GSH antioxidant activities results in female adolescent athletes and non-athletes. ** Significant differences between groups (*p* < 0.01).

Figure 1A,B show the differences in the catalase (CAT) and glutathione (GSH) between female adolescent athletes and non-athletes. According to independent t test analyses, there were significant differences between groups in terms of catalase (CAT) enzyme activity (t = 24.725; *p* = 0.001), with catalase activity being markedly higher in athletes compared to non-athletes. This corresponds to an exceptionally large effect size (Cohen’s d = 7.81), indicating a substantial enhancement of antioxidant defense associated with regular physical exercise.

However, there was no significant difference in glutathione (GSH) levels (t = 0.487; *p* = 0.629). Although GSH was slightly higher in athletes (Mean ± SD = 0.002 ± 0.001) compared to the non-athlete adolescent group (Mean ± SD = 0.002 ± 0.0004), the difference was not significant. Furthermore, the very small effect size (Cohen’s d = 0.15) suggests that regular physical activity exerts minimal influence on GSH levels. On the other hand, CAT activities were significantly higher when comparing the athlete group (Mean ± SD = 0.068 ± 0.011) and the non-athlete group (Mean ± SD = 0.003 ± 0.001). Importantly, Cohen’s d = 7.81, suggesting a strong enhancement of catalase activity following regular exercise.

**Figure 2:**
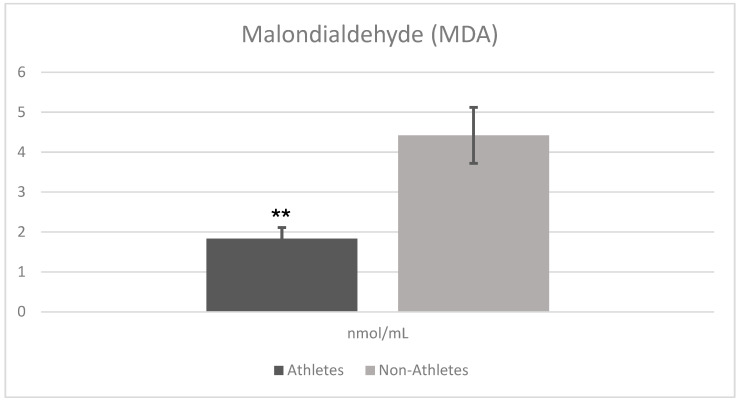
Comparison analysis of oxidative stress marker (MDA) results in female adolescent athletes and non-athletes. ** Significant differences between groups (*p* < 0.01).

Figure 2 shows the comparison analysis of oxidative stress marker (MDA) results in female adolescent athletes and non-athletes. The analysis results revealed that there was a statistically significant difference between the athlete and non-athlete groups in terms of malondialdehyde (MDA) levels. MDA levels were significantly lower in the athlete group (Mean ± SD = 1.834 ± 0.288) compared to the non-athlete group (Mean ± SD = 4.426 ± 0.709) (t = −15.133, *p* = 0.001). This difference reflects a very large effect size (Cohen’s d = 4.78), highlighting the practical importance of reduced oxidative stress in the athlete group.

To examine the relationship between CAT/GSH-mediated antioxidant defense and oxidative stress (MDA) levels, the Pearson Correlation Test was applied. The analysis results showed that there was no significant relationship between GSH and MDA levels (r = −0.071, *p* = 0.663). In contrast, a strong and statistically significant negative correlation was found between CAT activity and MDA levels (r = −0.900, *p* = 0.0001), demonstrating that increased catalase activity is closely associated with lower oxidative stress levels.

**Figure 3:**
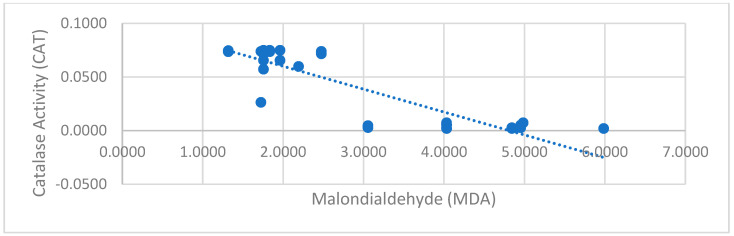
Correlation chart of catalase enzyme activity and malondialdehyde.

Moreover, the data indicate that GSH and CAT operate independently, as no significant correlation was found between their levels (r = −0.037, *p* = 0.819). This suggests that these two antioxidants play distinct roles in oxidative stress defense, which may have important implications for understanding their regulation in response to exercise (Figure 3).

## 4. Discussion

The effect of exercise on oxidative stress varies depending on the duration and frequency of application. Although the present study did not assess acute exercise responses, previous studies have reported that acute exercise may lead to a transient increase in oxidative stress due to rapid increases in cellular oxygen consumption and ROS generation. In contrast, chronic exercise may stimulate antioxidant defense systems, leading to long-term adaptive responses. These results are aligned with previous studies in the literature showing that chronic and acute exercise types produce different physiological effects. For example, in a study examining the oxidative stress responses of young Brazilian professional football players to exercise, da Costa and colleagues reported that free radicals formed during exercise caused oxidative damage to fatty acids in cell membranes. They reported that lipid peroxidation occurring during this process negatively affected muscle cell functions by weakening the structural integrity of cell membranes and, as a result, led to significant changes in malondialdehyde (MDA) levels [36]. Other studies with similar findings also have reported elevated MDA levels immediately after intense exercise or performance tests [37,38]; however, long-term physical activity has been associated with a subsequent decline in MDA concentrations. For example, Sopic et al. (2014) observed a decrease in MDA levels of 16 young soccer players after long-term trainings [39]. Similarly, the end of a year-long training program in middle-aged individuals reported that significant reduction MDA levels was observed [40]. Another study demonstrated that aerobic-based physical activity performed three days per week for 12 weeks significantly reduced MDA levels in sedentary individuals aged 32 to 50 years [23]. In another study, 24 weeks of aerobic exercise was found to reduce MDA levels [41]. Some studies have reported that long-term exercise does not lower MDA levels but helps keep them balanced by preventing them from rising. For example, in adolescent footballers with 12 weeks of endurance training showed no significant change in MDA levels [42]. The reduction in malondialdehyde (MDA) levels by long-term exercise is associated with adaptive biochemical mechanisms on oxidative stress balance. Chronic exercise accelerates the detoxification of reactive oxygen species (ROS) by increasing the expression of antioxidant enzymes, thus limiting the accumulation of MDA, the end product of lipid peroxidation. Increased oxygen consumption and mechanical stress due to exercise may initially trigger free radical production, causing a temporary increase in oxidative stress levels. However, repeated oxidative stimulation caused by regular exercise enables transcriptional reprogramming of endogenous antioxidant defense systems through activation of the Nrf2-ARE (Nuclear factor erythroid 2–related factor 2—Antioxidant Response Element) signaling pathway. This mechanism supports long-term adaptations to maintain redox homeostasis by increasing the expression of antioxidant genes such as SOD, CAT, GCLC, and HO-1. As a result, a decrease in MDA levels, an indicator of lipid peroxidation, is observed in individuals who exercise, which contributes to the suppression of oxidative damage and the reduction of the risk of cardiovascular and neurodegenerative diseases [43,44,45]. However, the severity and duration of oxidative stress play a critical role in determining which antioxidant defense mechanism will become dominant. While Nrf2-ARE-mediated adaptive responses come to the fore in mild oxidative stress conditions, when hydrogen peroxide (H_2_O_2_) is formed at high concentrations, the rapid and direct effects of antioxidants such as catalase (CAT) and glutathione (GSH) come to the fore in the first step of cellular detoxification. Indeed, as reported by Radi et al. (1991), the catalase enzyme is more significantly activated at high H_2_O_2_ concentrations and becomes the primary defense element [46]. Accordingly, it can be said that exercise-induced oxidative stress responses are managed by time- and dose-dependent, complementary defense systems in both acute and chronic contexts.

Catalase (CAT) and glutathione (GSH) and interplay between these systems is crucial in regulating oxidative stress. The progressively increasing metabolic demand elevates cellular oxidative load, thereby triggering adaptive response mechanisms. In this adaptation process, catalase (CAT) and glutathione (GSH) antioxidants play a critical role. CAT is one of the main antioxidant enzymes that reduces oxidative stress by breaking down hydrogen peroxide (H_2_O_2_) into water and oxygen. GSH is oxidized by glutathione peroxidase (GPx) to glutathione disulfide (GSSG) and then returned to its reduced form (GSH) using NADPH via the enzyme glutathione reductase (GR). This process plays a vital role in maintaining cellular redox balance and managing oxidative stress [47,48,49]. In our study, CAT activity significantly increased at trained female adolescent athletes, while GSH levels were statically significantly unchanged. This result is consistent with some research showing that chronic exercise increases enzymatic antioxidant capacity, although specific adaptations may vary. For instance, adolescent runners assessed before and after a year of training exhibited increased CAT activity with no change in GSH levels [50]. This finding supports the results obtained in our study. Similarly, another study on adolescent swimmers reported that 8 weeks of training was insufficient to alter antioxidant markers, whereas 16 weeks of training led to simultaneous increases in both CAT and GSH levels [51]. These findings highlight that training duration and intensity are key determinants of antioxidant activation. The stability of GSH levels, despite the increase in CAT activity, suggests a dynamic regulatory mechanism within the antioxidant defense system. Some studies have reported inverse relationships between these both systems. For example, six months of football training in adolescent males led to increased CAT levels but decreased GSH levels [17]. On the other hand, in a study on swimmers, GSH levels increased while CAT levels remained unchanged after in similar training time [35]. The possible mechanism explaining these discrepancies could be that CAT and GPx respond to different levels of H_2_O_2_ accumulation [5]: CAT primarily decomposing high H_2_O_2_ concentrations, while GPx is activated at lower H_2_O_2_ levels. As GPx plays a key role in utilizing GSH to neutralize ROS, a shift toward greater CAT activity could explain why some studies observe unchanged or decreased GSH levels despite ongoing oxidative stress [46]. There is evidence in the literature that catalase provides a rapid and effective detoxification response, especially at high H_2_O_2_ levels, while the GSH system plays a more buffering role that maintains the redox balance [46,52]. Ji (1999) reported that although GSH is oxidized during exercise via the activity of glutathione peroxidase (GPx), there is generally no significant change in the GSH:GSSG ratio due to the reduction of GSSG back to GSH via the glutathione reductase enzyme [52]. In this context, the fact that only GSH levels were examined in the present study provides a limited view of the glutathione system. However, the results obtained provide important biological insights in terms of the stability of the GSH system and the predominant activation of CAT in response to exercise-induced oxidative stress.

In our study, the effect sizes obtained for MDA and CAT enzyme activity parameters were observed to be unusually large. Although uncommon, such large effect sizes may occasionally occur due to specific biological or experimental conditions. For example, Serdar et al. (2021) reported a “huge” Cohen’s d value when comparing total cholesterol levels between two groups, despite the absolute mean difference being as small as 1.3 mmol/L [34]. Similarly, previous research has documented huge effect sizes in studies involving pain perception [53] and in experiments characterized by low within-group variability [54]. In our case, the combination of remarkably low within-group variance, significant group differences, and inherently limited biological variability likely contributed to these very large effect sizes. Supporting this, Yagin et al. (2024) highlighted that, under certain conditions such as variation in educational background or the intensity of interventions, exceptionally large effect sizes can be observed [55]. This is consistent with our findings, as the elevated effect sizes may be attributed to the superior physiological adaptation capacity of the athlete group included in our sample.

A strong negative correlation between CAT and MDA levels (r = −0.900) supports our hypothesis that increased antioxidant enzyme activity mitigates oxidative stress. This finding reveals that the antioxidant systems of participants with low oxidative stress levels are more active, and that the catalase enzyme is especially more active in this group. Such a strong negative correlation may also be due to the homogeneity of the athlete group, as a relatively uniform physiological adaptation and reduced within-group variability may have amplified the relationship between CAT activity and MDA levels. Although direct correlation analysis has not been conducted in CAT and MDA in the literature, the negative correlation in the current study is indirectly supported by some previous studies. One study demonstrated that in exercise-based cycling trials with sedentary adults, MDA levels increased as intensity and exercise time, while CAT activity declined [56]. In another study, a short-term training program applied to soccer players led to increased MDA levels and decreased CAT activity [54]. These studies, although not directly examining correlation, they suggest that CAT and MDA levels are negatively related, as observed in our study. Although numerous studies have investigated the effects of exercise on oxidative stress markers, relatively few have examined the CAT–MDA correlation [25,40,56,57,58,59,60]. In this context, in the study conducted by Djordjevic et al. (2011), oxidative stress levels of trained and untrained young handball players were compared, and it was found that antioxidant defense systems were stronger in individuals who exercised regularly, but MDA levels varied depending on the intensity of the exercise. This shows that exercise creates adaptive mechanisms, but oxidative stress can increase if a certain threshold is exceeded [25]. Moreover, Sureda et al. (2005) evaluated the relationship between oxidative stress markers and endogenous antioxidant defenses during exercise to the point of exhaustion [59]. The study reported that MDA levels increased with increased exercise duration, whereas antioxidant enzymes such as catalase (CAT) tended to be depleted. Moreover, our research results revealed that GSH and CAT antioxidant systems work independently of each other, and there is no significant correlation between them. The increase in only CAT activity after chronic exercise was explained by the specific and rapid adaptation of enzymatic antioxidants to chronic exercise. On the other hand, since GSH has more extensive and indirect mechanisms in antioxidant defense, its adaptation to chronic exercise occurs more slowly and does not show a significant change compared to antioxidants that directly target ROS, such as CAT. The findings obtained by Tong and his colleagues, in a study conducted on adolescent runners after one year of training, support the results of our research and the hypothesis we proposed [50].

This study has several strengths. It is one of the rare studies examining the correlation between oxidative stress and antioxidant activity and these parameters in adolescent female athletes. Furthermore, it provides important insights into the physiological adaptations of long-term training, making a valuable contribution to the field of sports science and exercise physiology. Additionally, the study participants were highly specific in terms of age, sex, and training background, allowing for a focused analysis of exercise-induced oxidative stress responses. The biochemical analyses were conducted with high precision, ensuring reliable and reproducible results. However, certain limitations should be acknowledged. This study did not apply a controlled training intervention but instead analyzed participants based on their existing training routines. Although all athletes had been training regularly for at least three years, individual variations in training intensity, type, and volume may have influenced the results. It is also unknown whether female athletes are equally affected by physical effort. Training in the same sport is different for female athletes playing in different positions. Additionally, dietary intake and nutritional status were not controlled, which could have affected antioxidant defense parameters and oxidative stress markers. Another notable limitation is that the menstrual cycle phase of the participants was not assessed. There are controversial views in the literature regarding the significant effects of the menstrual cycle on oxidative stress and antioxidant defense mechanisms due to hormonal changes. Therefore, failure to consider this factor in the study may have resulted in interindividual differences in biochemical parameters. Moreover, this study focused only catalase (CAT) and glutathione (GSH) as antioxidant markers, but other antioxidants such as superoxide dismutase (SOD) and glutathione peroxidase (GPx) were not included.

## 5. Conclusions

In conclusion, oxidative stress is a key factor in exercise physiology, representing the dynamic balance between ROS production and antioxidant defense mechanisms. This study demonstrates that long-term exercise reduces oxidative stress and improves specific components of the antioxidant defense system, as reflected by increased CAT activity and reduced MDA levels, in adolescent girls. The use of MDA as a marker of lipid peroxidation provides reliable evidence of oxidative damage, while CAT and GSH were selected due to their fundamental roles in enzymatic and non-enzymatic antioxidant defenses. Our findings suggest that regular physical activity contributes to the maintenance of cellular integrity and redox homeostasis during adolescence. Interestingly, the marked increase in CAT but not GSH may be partially explained by activation of the Nrf2-ARE pathway, a transcriptional regulator that promotes antioxidant enzyme expression in response to oxidative stress. These findings highlight the importance of promoting exercise habits to support oxidative balance and health in this critical developmental period.

However, the variability in GSH responses suggests that its regulation by chronic exercise remains unclear and may be influenced by factors such as training background, intensity, nutrition, or hormonal status. Future research should systematically examine how long-term exercise protocols—varrying in intensity and duration, and focusing on either aerobic or anaerobic mechanisms—affect oxidative stress adaptations individually, while also considering the role of dietary antioxidants in modulating these effects. In addition, it seems necessary to design new studies that compare the responses of different age groups to physical loads. The influence of hormonal changes, particularly the antioxidant-related effects of estrogen, should be considered when investigating oxidative stress adaptations, as they may contribute to sex-specific differences during adolescence. It would also be worthwhile to determine the maximum estrogen concentrations during the menstrual cycle. Recognizing individual differences such as age, training status, and metabolic variability is essential for tailoring effective and safe exercise interventions. Finally, future studies employing longitudinal and well-structured cohort designs are warranted to clarify the long-term and sex-specific adaptations of oxidative stress responses during adolescence. Such insights could inform targeted strategies to optimize recovery, performance, and long-term health outcomes in young athletes.

## Figures and Tables

**Table 1:** Baseline characteristics of the analyzed sample (n = 40).

Parameters	Athletes (n = 20)	Non-Athletes (n = 20)	Effect Size (d)	*p*-Value
	M ± SD	M ± SD		
Age (years)	16.65 ± 0.67	16.80 ± 0.69	Medium	0.49
Weight (kg)	59.75 ± 5.50	60.15 ± 4.23	Large	0.79
Height (cm)	165.50 ± 6	159.95 ± 4	Large	<0.001
BMI (kg/m^2^)	21.81 ± 1.50	23.51 ± 1.60	Large	<0.001

M = mean; SD = standard deviation.

## Data Availability

The original contributions presented in the study are included in the article; further inquiries can be directed to the corresponding author.
